# Association between Healthy Eating Index-2015 and Age-Related Cataract in American Adults: A Cross-Sectional Study of NHANES 2005–2008

**DOI:** 10.3390/nu15010098

**Published:** 2022-12-25

**Authors:** Jingxin Zhou, Lixia Lou, Kai Jin, Juan Ye

**Affiliations:** 1 Department of Ophthalmology, School of Medicine, The Second Affiliated Hospital of Zhejiang University, Hangzhou 310009, China; 2 Zhejiang Provincial Key Lab of Ophthalmology, Hangzhou 310009, China

**Keywords:** cataract, healthy eating index-2015, healthy eating pattern, NHANES, epidemiology

## Abstract

Background: Healthy Eating Index-2015 (HEI-2015), a healthy eating pattern proposed by Dietary Guidelines for Americans, is proven to be protective against various diseases. However, whether it is associated with age-related cataracts is unknown. Methods: This population-based cross-sectional study included 6395 participants from the National Health and Nutrition Examination Survey (NHANES) in the cycles from 2005 to 2008. HEI-2015 was calculated from 24-h dietary recall interviews, ranging from 0 to 100, and higher HEI-2015 represents better diet quality. Age-related cataract was generated from the questionnaire. The association between HEI-2015 and cataract was assessed with logistic regression models. Propensity score weighting, restricted cubic spline, and subgroup analysis were conducted to further explore the relationship. Results: 6395 participants were included in the study, with a mean [standard deviation (SD)] age of 48.7 (15.3) years and 3115 (48.7%) being male. HEI-2015 was negatively associated with cataracts after adjusting all covariates included, both as a continuous variable [odds ratio (OR): 0.991, 95% confidence interval (CI): 0.984–0.997, *p* = 0.006] and quartiles with the highest quartile compared to the lowest (OR: 0.739, 95% CI: 0.559–0.980, *p* = 0.035). After propensity score weighting, the association remained significant. Restricted cubic spline revealed no non-linear relationship (*p* for non-linearity 0.085). Subgroup analysis showed that there were no interaction effects. Conclusions: Adherence to the healthy eating pattern, HEI-2015, was associated with a lower risk of age-related cataracts.

## 1. Introduction

Cataracts, opacification of the lens, is one of the most common vision impairment and blindness-causing conditions among older adults worldwide [1,2]. Aging is the leading cause of cloudy lenses in most cases, namely age-related cataract. It is associated with many factors, such as smoking, diabetes, and sunlight exposure [3]. Although cataract surgery can effectively improve the vision of cataract patients, it is cost-prohibitive, and there is an insufficient number of qualifiable surgeons in some developing countries [4]. Identifying modifiable risk factors and managing them is of great help in alleviating the health and economic burden of cataracts.

Diet is a modifiable behavior not only crucial for energy supply but also associated with diseases [5]. Several nutrients are associated with cataracts, including carbohydrates, vitamins, and carotenoids, etc. [6,7]. However, nutrients are not consumed separately but in sophisticated food combinations. Thus, dietary pattern, an overall reflection of food-consuming structures, is able to reflect authentic diet practices and yield actionable dietary recommendations [8,9].

Healthy Eating Index-2015, a diet pattern measure, is commonly used to evaluate diet quality according to the healthy eating pattern proposed by the 2015–2020 Dietary Guidelines for Americans (DGA) [10], and it was also recommended in 2020–2025 DGA [11]. HEI-2015 has been proven to be associated with cardiovascular diseases, metabolic disorders, cancer, etc. [8,12,13,14,15,16]. However, there is little knowledge about the relationship between HEI-2015 and cataract risk.

In this article, we used data from National Health and Nutrition Examination Survey (NHANES) 2005–2008 to conduct a cross-sectional study investigating the association between HEI-2015 and cataract risk. We hypothesized that higher HEI-2015 is associated with lower cataract risk.

## 2. Materials and Methods

### 2.1. Data Source and Study Population

NHANES is a large nationally representative survey designed to assess the health and nutritional status of the American population, conducted by the National Center for Health Statistics of the U.S. Centers for Disease Control and Prevention [17]. Survey data in NHANES were organized in a biannual form. 

**Figure 1:**
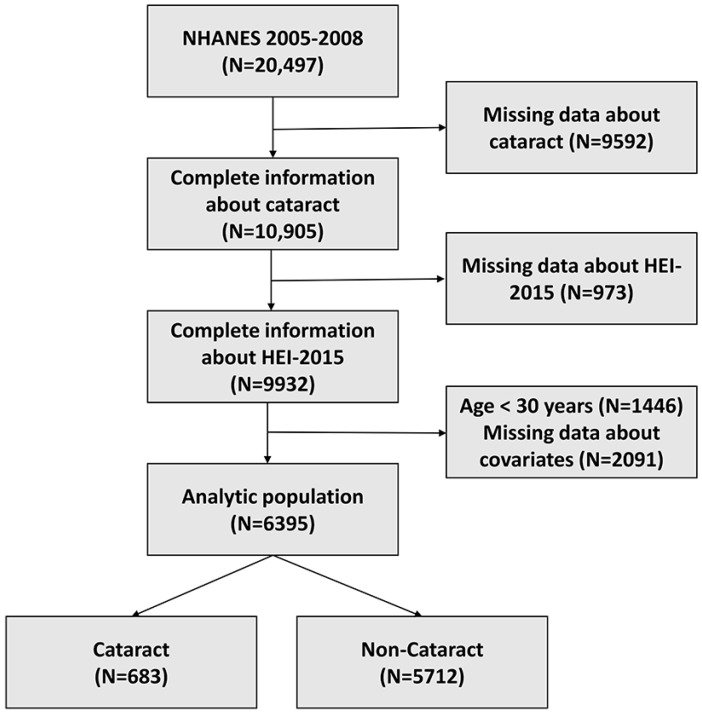
Flow chart of the study population.

We utilized data from 2 consecutive survey cycles (2005–2006 and 2007–2008) about cataracts. Of all 20,497 participants in NHANES 2005–2008, we excluded those without complete information on cataracts (*n* = 9592) and diet (*n* = 973). Further, we excluded participants under 30 years old (*n* = 1446) without complete information on other covariates (*n*= 2091). Finally, 6395 subjects were included in the analytic population. The process of participant selection is summarized in Figure 1.

### 2.2. Cataract Assessment

Consistent with other epidemiological research, a cataract operation was used as a surrogate for a cataract [18]. Cataract operation was determined by asking participants the question, “Have you ever had a cataract operation?” (VIQ071), with answers “yes” or “no”. If the answer was “yes”, the participant was diagnosed with a cataract. 

### 2.3. Healthy Eating Index-2015 Assessment

Dietary information in NHANES was obtained from the What We Eat in America (WWEIA) program conducted by the United States Department of Agriculture (USDA). Dietary data were collected from 24-h dietary recalls using the Automated Multiple-Pass Method (AMPM) provided by USDA [19]. The calculations of food groups, nutrients, and energies were conducted by the USDA Food Patterns Equivalence Database (FPED) [12].

HEI-2015 is a continuous score from 0 to 100, with a higher number representing better adherence to the recommended diet pattern by DGA [10,11], which is seen as better diet quality [20]. HEI-2015 consists of 13 food components: 9 adequacy components and 4 moderation components. Of the 9 adequacy components, 6 of them are with 0–5 points (total fruits, whole fruits, total vegetables, greens, beans, total protein foods, seafood, and plant proteins), and 3 of them are with 0–10 points (whole grains, dairy, fatty acids). Four moderation components (sodium, refined grains, added sugars, and saturated fats) are 0–10 points each. The components’ points were summed to compute the final HEI-2015 score [21]. It should be noted that the calculation was not based on the absolute amount of components but on the energy density per 1000 kcal, which can be extracted from FPED. In our study, following previous studies, dietary information was extracted from the Total Nutrient Intakes of the First Day (DR1TOT_I) in NHANES and further transformed and calculated with the help of data from FPED [12,21,22,23]. 

### 2.4. Covariates Assessment

According to previous epidemiological studies concerning cataracts, potential confounding factors studied in the current work included sociodemographic factors (gender, age, race, education level, marital status, and economic situation), physical measures (body mass index (BMI)), lifestyle factors (alcohols usage, smoking), and comorbidities (hypertension, hyperlipidemia, diabetes mellitus) [18,24]. 

Sociodemographic factors were drawn from self-reported questionnaires, including gender (male, female), age (continuous), race (non-Hispanic white, non-Hispanic black, Mexican American, other), education (less than high school, high school or higher), marital status (married or living with a partner, unmarried, or other), economic situation (family income poverty ratio <1.00, or ≥1.00). BMI was calculated as weight (kg) divided by height squares (m^2^) using information from body measurement examinations and further categorized into 3 classes (<18.5, 18.5~25, >25 kg/m^2^). 

Lifestyle factors were obtained from self-reported questionnaires. Alcohol usage was calculated and categorized as lifetime abstainer (fewer than 12 drinks in a lifetime), former drinker (at least 12 drinks in lifetime but no drinks in past year), current drinker ≤ 3 drinks/week, and current drinker > 3 drinks/week [25]. Smoking was divided into 3 categories: non-smoker (never smoked or smoked < 100 cigarettes in a lifetime), former smoker (smoked at least 100 cigarettes in a lifetime but had quit smoking by the time of interview), and current smoker [26]. 

Comorbidities studied in this study included hypertension, hyperlipidemia, and diabetes mellitus. Participants were considered to have hypertension if they had been told by their doctors that they had hypertension, if they were taking anti-hypertension drugs, or their systolic blood pressure was 140 mmHg or greater, or diastolic blood pressure was 90 mmHg or greater. Diagnosis of hyperlipidemia was made if participants were told they had hyperlipidemia or were taking cholesterol-lowering drugs or their total cholesterol was no less than 240 mg/dL during the NHANES blood test. The presence of diabetes mellitus was determined if participants were told they had diabetes mellitus, were taking glucose-lowering drugs, or using insulin injections, or their glycosylated hemoglobin (%) was 6.5% or greater during the NHANES test.

### 2.5. Statistical Analysis

Continuous variables were described using mean ± standard deviation (SD), and categorical variables were presented as numbers and percentages. HEI-2015 was analyzed as continuous and categorical variables based on quartiles. Variables were compared using Student’s *t*-test or Rao-Scott Pearson χ^2^ test. To investigate the association between HEI-2015 and cataract, three logistic regression models were established. Variance inflation factors (VIFs) were calculated to examine the possible multi-collinearity of all variables in logistic models, and we found that all VIFs were less than 2, meaning there was no multi-collinearity among the studied variables. Given that some population characteristics were significantly different in subjects with different HEIs, as in Supplementary Table S1, propensity score weighting (PSW) using inverse probability weight, a common PSW method, was calculated, and PSW-weighted multivariate logistic regression models were established to further control confounders. HEI-2015 is a continuous variable, and the calculation of propensity score for continuous variables followed the methods proposed in [27]. A restricted cubic spline model with 3 knots was utilized to explore potential non-linear associations. The choice of knot number 3 was based on minimizing the Akaike information criterion (AIC) statistic. RCS analysis was adjusted for all covariates. Subgroup analyses based on all covariates were conducted to investigate differences in subgroups and explore latent interaction effects. 

The statistical analysis and visualization were conducted using R (version 4.1.1, R Foundation for Statistical Computing, Vienna, Austria). All statistical tests were two-tailed with a *p*-value of 0.05 or smaller as significant.

## 3. Results

### 3.1. Study Population Characteristics

A total of 6395 participants were included in the study population, with a mean age of 48.7 years, 3115 (48.7%) males and 3280 (51.3%) females. The characteristics are summarized in Table 1. Participants with cataract surgery were more likely to be older unmarried females with lower education levels and relatively better family economic situations. Patients with a history of smoking or alcohol usage were found to be more likely to have cataracts. Patients with hypertension, hyperlipidemia, and diabetes mellitus were also at risk of developing cataracts. However, contrary to our hypothesis, it can be found in Table 1 that participants with cataracts tended to have higher HEI-2015 scores. Multivariate analysis is needed.

### 3.2. Association of HEI-2015 and Cataract Risk Using Logistic Regression

Results of logistic regression models for the association between HEI-2015 and cataract risk are shown in Table 2. HEI-2015 was positively associated with cataract risk in the non-adjusted model (Model 1) both as a continuous variable (OR: 1.019, 95% CI: 1.014–1.024, *p* < 0.001) and as a categorical variable with the highest quartile compared to the lowest (OR: 2.121, 95% CI: 1.692–2.671, *p* < 0.001). However, the results in the minimally adjusted model (Model 2) and fully adjusted model (Model 3) were the opposite. HEI-2015 was significantly negatively associated with cataracts in Model 2 (as a continuous variable: OR 0.991, 95% CI 0.984–0.997, *p* = 0.002; as quartile: OR 0.751, 95% CI 0.569–0.940, *p* = 0.044) and Model 3 (as a continuous variable: OR 0.991, 95% CI 0.984–0.997, *p*= 0.006; as quartile: OR 0.739, 95% CI 0.559–0.980, *p* = 0.035). Compared to univariate regression, the multivariate regression model is more reliable. Namely, HEI-2015 is negatively associated with cataract risk in the current study. However, further analysis should be done to disentangle the contradiction.

### 3.3. Association of HEI-2015 and Cataract Using Propensity Score Weighted Regression

As is shown in Table 1, population characteristics between participants with and without cataracts were significantly different among all covariates. To further control the confounding effects, propensity score weighting (PSW) was adopted using the inverse probability weight method, followed by PSW-weighted multivariate logistic regression analysis. Participant characteristics comparison before and after weighting were summarized in Table S1 and Table S2, respectively, from which we could draw the conclusion that PSW effectively reduced the variations of covariates among different HEI-2015 quartiles. 

The results of PSW-weighted regression models were shown in Table 3. HEI-2015 was negatively associated with cataract risk as a continuous variable in the non-adjusted model (OR: 0.991, 95% CI: 0.985–0.996), *p* < 0.001), minimally-adjusted model (OR: 0.992, 95% CI: 0.984–0.996, *p* = 0.002), and fully-adjusted model (OR: 0.990, 95% CI: 0.984–0.995, *p* = 0.002). Compared to the lowest HEI-2015 quartile, the highest HEI-2015 quartile was negatively associated with cataracts in the non-adjusted model (OR: 0.748, 95% CI: 0.605–0.923), *p* = 0.007), minimally-adjusted model (OR: 0.747, 95% CI: 0.576–0.967, *p* = 0.027), and fully-adjusted model (OR: 0.744, 95% CI: 0.572–0.967, *p* = 0.027). Results from PSW-weighted regression revealed that in all three models, HEI-2015 was significantly negatively associated with cataracts, inferring that the contradicted results of unweighted regression were due to confounding effects.

### 3.4. Association of HEI-2015 Components and Cataract

Each component score of HEI-2015 was treated as a single variable, and logistic regression models were established to investigate the association of HEI-2015 components and cataracts. Results are summarized in Table 4. We found that among all these components, total fruits (OR: 0.947, 95% CI: 0.903–0.993), *p* = 0.027), whole fruits (OR: 0.948, 95% CI: 0.907–0.991), *p* = 0.016), whole grains (OR: 0.966, 95% CI: 0.937–0.995), *p* = 0.024), and refined grains (OR: 0.958, 95% CI: 0.932–0.985), *p* = 0.002) were significantly associated with cataract risk. 

### 3.5. Investigation of Non-Linear Association Using Restricted Cubic Spline

**Figure 2:**
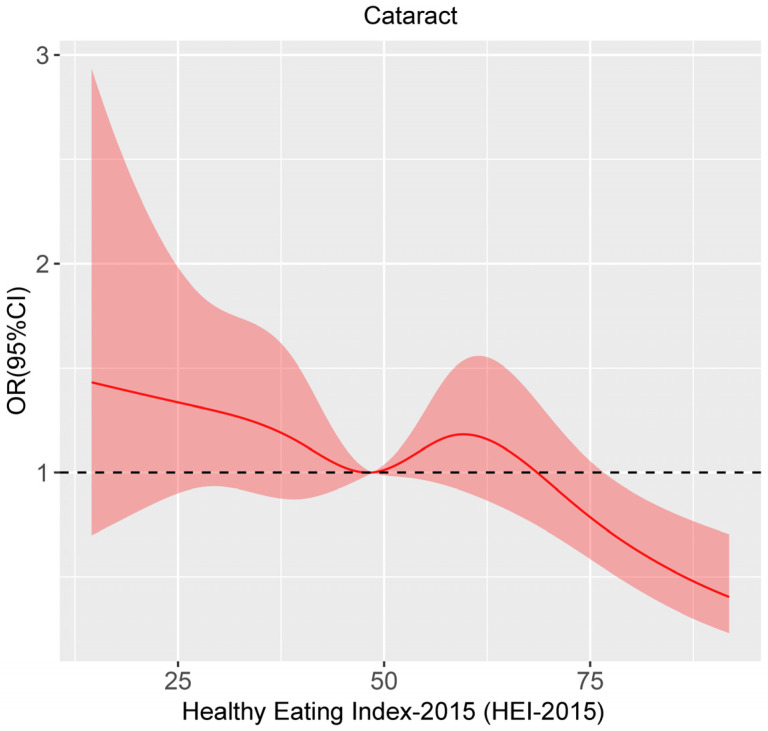
Restricted cubic spline analysis of the association between HEI-2015 and cataract.

To further test the presence of non-linear association between HEI-2015 and cataract, three-knot restricted cubic spline was adopted. The *p*-value for non-linearity test was 0.085, meaning that there is no significant non-linear relationship between HEI-2015 and cataract. As is shown in Figure 2, the curve presents an overall declining trend, indicating that there is a negative association between HEI-2015 and cataract.

### 3.6. Subgroup Analyses

**Figure 3:**
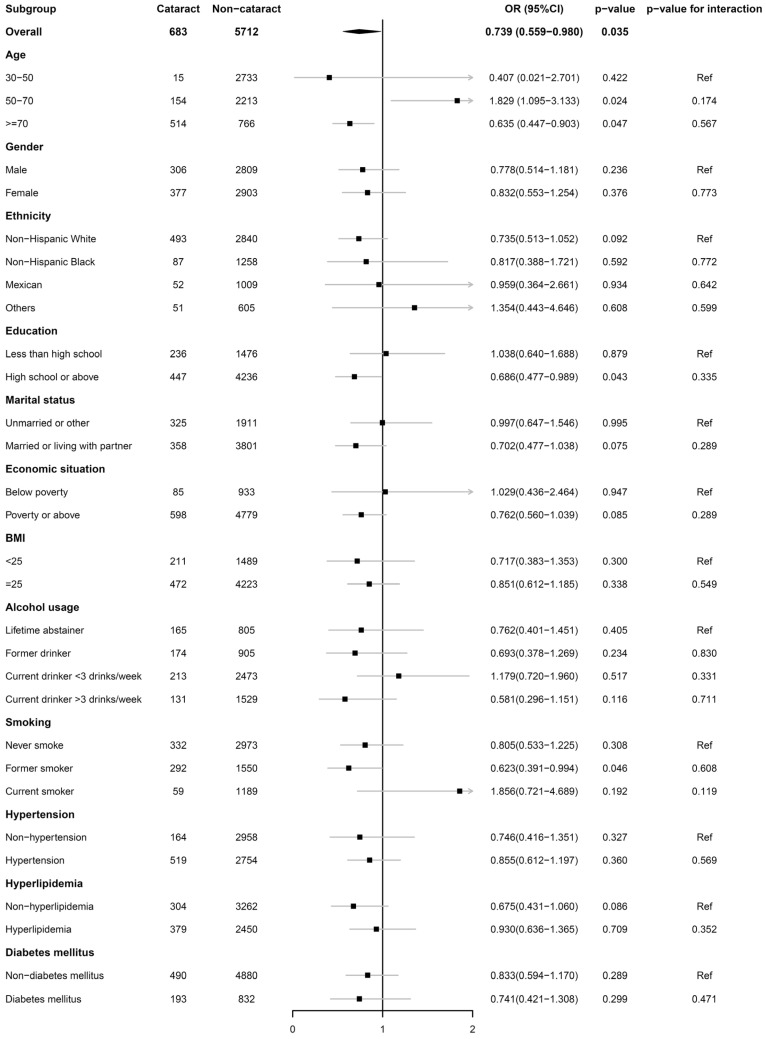
Subgroup analysis of the association of HEI-2015 with cataract.

Subgroup analyses were conducted on all covariates using fully-adjusted logistic regression model. Results are summarized in Figure 3. For most groups of participants, HEI-2015 remained a negative association with cataract. For participants with hypertension, hyperlipidemia and diabetes mellitus, it was also negative.

## 4. Discussion

In this cross-sectional study, we examined the association between HEI-2015 and the prevalence of cataracts by extracting 6395 participants’ data from NHANES. Results demonstrated that there was a significantly negative association between HEI-2015 and cataract surgery in a fully-adjusted multivariate analysis, which means that subjects with a healthy eating pattern, according to the DGA 2015, are less likely to go through cataract surgery. Further analysis, including propensity score, weighted regression, non-linearity test, and subgroup analysis, additionally confirmed this conclusion.

The DGA is updated every five years, and HEI-2015 was proposed in DGA 2015. In the latest DGA 2020, HEI-2015 continues to be recommended as a healthy eating pattern. The relationship between different versions of HEI and cataract has been investigated in previous research, most of which reported a protective effect. In 2004, Moeller et al. conducted a prospective study using information from 479 Nurses’ Health Study participants and found that with a 9- to 11-year follow-up, participants with the highest HEI-1995 quartile were less likely to have nuclear cataracts than those in the lowest category (OR: 0.47, 95% CI: 0.26–0.84) [28]. A cross-sectional study from India also confirmed the protection of HEI-1995 against cataracts [29]. In 2010, Mares et al. analyzed 1808 participants from the Women’s Health Initiative (WHI), prospectively, and found that HEI-1995 was protective against the development of nuclear cataracts (high versus low quintile, OR: 0.63, 95% CI: 0.43–0.91), while there was no significant negative association between HEI-2005 and lens opacity (high versus low quintile, OR: 1.12, 95% CI: 0.78–1.59) [30]. The authors thought it was due to the recommendations of high oil intake in DGA 2005 [30]. In 2019, Ava et al. investigated the relationship between the Australian version of HEI and incident cataract from 2173 participants in the Blue Mountains Eye Study [24]. They found that baseline Australian-HEI was not associated with any kind of cataract in the whole analytic population, but for participants with a BMI lower than 25 kg/m^2^, increasing HEI was associated with decreased risk of nuclear cataract (per unit increased HEI, OR: 0.90, 95% CI: 0.81–0.99), indicating the latent interaction effects of BMI and HEI [24]. Overall, researchers tended to believe that healthy eating patterns according to DGAs are beneficial for maintaining of lens transparency. All these studies emphasized the importance of a healthy diet for the prevention of lens opacification. However, to our knowledge, there was no study focusing on the latest HEI-2015 and cataract risk. 

It is necessary to study HEI-2015, the latest version, both theoretically and practically. Theoretically, there are unignorable alterations in HEI-2015. The main changes between HEI-2015 and former HEI-2010 include three aspects: (1) saturated fat and added sugars are taken as single components, reflecting the explicit recommendations to limit intakes of these specific nutrients by DGA 2015; (2) alcohol energy is not calculated as a separate component but counted in the total energy, making it suitable for separate assessment or multivariate analysis, as in our study; (3) legumes are counted in both vegetables and protein foods, instead of in either vegetables or protein foods, aiming to alleviate the complex computation of HEI and difficulty capturing protein variety [20]. These changes made it necessary to conduct the research. Practically, HEIs provided by DGAs were broadly used by different levels of the food environment to assess the diet quality, from federal food distribution program to grocery store [20,31,32,33]. Investigation of HEI-2015 is helpful to provide evidence of the relationship between current healthy diet and vision health. 

Oxidative stress has long been seen as an important pathological process of cataract formation [34,35,36]. In the past decades, epidemiologists have found several types of antioxidant-rich foods are protective against cataracts, including fruits and vegetables, vitamins, carotenoids, and antioxidant supplements, etc. [6,7,30,37,38,39,40,41,42,43,44,45,46,47,48,49,50]. Through analyzing the components of HEI-2015, we also found that higher intake of fruits, whole grains and lower consumption of refined grains was associated with lower risk of cataract. Diet with a high glycemic load is found to be a risk factor in cataract formation [51,52,53]. Additionally, the antioxidant capacity of the whole diet is inversely associated with cataract risk [54,55], and diet quality assessed by HEI has been proven to be positively correlated with an antioxidant capacity [56]. Higher HEI is also associated with lower oxidative stress biomarkers [57]. Taking all of these into consideration, we believe that the low oxidative stress brought by a healthy eating pattern is beneficial for maintaining lens transparency.

In the current study, results from univariate and multivariate regression were controversial, which is well worth discussing. We think the dilemma here can be explained by a statistically uneven population. It can be seen in Supplementary Table S1 that for participants with different HEI-2015 quartiles, the sociodemographic, lifestyle, physical measurement, and comorbidity factors were most significantly different. In situations such as this, univariate models cannot yield reliable results. Using PSW to reduce these variances, as is in Supplementary Table S2, the univariate regression model could also generate results in the same direction as multivariate analysis. Therefore, we tend to believe the discrepancy was caused by confounding effects. We thought that the main confounder was age, as in the minimally-adjusted model in Table 2, when the contradiction was resolved. Participants with cataract surgery were older and had more comorbidities compared to those without. They possibly chose to eat healthier, resulting in a positive correlation.

Subgroup analysis also revealed that the contradicted results were derived from participants’ age. We believe that the positive association in age groups can be explained by the fact that older people tend to eat healthier and, thus, have higher HEI-2015 scores. The Pearson correlation coefficient between HEI-2015 and age was 0.211 with a *p*-value less than 0.001, which meant that in our study population, HEI-2015 was mildly but significantly correlated with age in a positive way. In multivariate regression models controlling age, the positive relationship was fixed, but in subgroup analysis, where age was used for stratification and not included in regression models, the controversial results reappeared. What is more, in subjects aged over 70, the results should be interpreted with care as potential survival bias may affect the authenticity.

Cataract surgery was used as a surrogate for cataract in this article, as there is no lens examination in NHANES. There is also an epidemiological study using a similar method [18]. However, the differences between them cannot be neglected. First, cataract surgery depends on many factors, including cataract grading, visual acuity, ophthalmologists’ decision, patients’ choice, etc. Only patients with sufficient financial conditions would have the opportunity to go through that operation, and financial conditions would also affect participants’ health awareness and diet quality. In our study, the financial conditions were taken as covariates to reduce the potential confounding effects. Second, cataract surgery represented a relatively advanced stage of cataract. Association between the earlier-stage lens opacification and HEI-2015 could not be investigated using information from NHANES. Third, with the information from cataract surgery, we could not distinguish which participants had which type of cataract. 

The advantages of the current study lie in the novelty of the topic, the relatively large sample size, and the comprehensive statistical methods. There are also several limitations. First, this is a cross-section study, and we cannot drive a causality conclusion from it. Second, cataract surgery was used as a surrogate for cataract, causing latent problems discussed before. Third, there are still residual confounding factors that were not discussed in the study. Nonetheless, we used PSW-weighted multivariate logistic regression models to deal with confounders, and the results were similar, verifying the reliability of the conclusion. Fourth, the conclusions were drawn from a national survey in America, so they may not be generalizable in other racial populations.

## 5. Conclusions

In this cross-sectional study, including 6395 participants from a large nationally representative survey, we found a significantly negative association between HEI-2015 and cataract risk. The study implied that high diet quality, according to DGA, is negatively associated with cataract risk. Further PSW analysis, subgroup analysis, and non-linearity test also confirmed the results. However, large prospective studies are needed to examine the conclusion to confirm the causal relationship between HEI-2015 and cataract development risk.

## Figures and Tables

**Table 1:** Characteristics of participants stratified by cataract from NHANES 2005–2008.

	All	Non-Cataract	Cataract	*p* Value
Number	6395	5712 (89.3)	683 (10.7)	
Gender (N, %)				0.034
Male	3115 (48.7)	2809 (49.2)	306 (44.8)	
Female	3280 (51.3)	2903 (50.8)	377 (55.2)	
Age (years, mean (SD))	48.7 (15.3)	51.7 (14.1)	74.4 (9.0)	<0.001
Race (N, %)				<0.001
Non-Hispanic White	3333 (52.1)	2840 (49.7)	493 (72.2)	
Non-Hispanic Black	1345 (21.0)	1258 (22.0)	87 (12.7)	
Mexican American	1061 (16.6)	1009 (17.7)	52 (7.6)	
Other	656 (10.3)	605 (10.6)	51 (7.5)	
Education (N, %)				<0.001
Less than high school	1712 (26.6)	1476 (25.8)	236 (34.6)	
High school or above	4683 (73.4)	4236 (74.2)	447 (65.4)	
Marital status (N, %)				<0.001
Unmarried or other	2236 (35.0)	1911 (33.5)	325 (47.6)	
Married or living with partner	4159 (65.3)	3801 (66.5)	358 (52.4)	
Economic situation (N, %)				<0.001
Below poverty	1018 (15.9)	933 (16.3)	85 (12.4)	
Poverty or above	5377 (84.1)	4779 (83.7)	598 (87.6)	
BMI (N, %)				0.018
<18.5	83 (1.3)	75 (1.3)	8 (1.2)	
18.5~25	1617 (25.3)	1414 (24.8)	203 (29.7)	
≥25	4695 (73.4)	4223 (73.9)	472 (69.1)	
Alcohol usage (N, %)				<0.001
Lifetime abstainer	970 (15.2)	805 (14.1)	165 (24.2)	
Former drinker	1079 (16.9)	905 (15.8)	174 (25.5)	
Current drinker ≤ 3 drinks/week	2686 (42.0)	2473 (43.3)	213 (31.2)	
Current drinker > 3 drinks/week	1660 (26.0)	1529 (26.8)	131 (19.2)	
Smoking (N, %)				<0.001
Never smoke	3305 (51.7)	2973 (52.0)	332 (48.6)	
Former smoker	1842 (28.8)	1550 (27.1)	292 (42.8)	
Current smoker	1248 (19.5)	1189 (20.8)	59 (8.6)	
Hypertension (N, %)				<0.001
No	3122 (48.8)	2958 (51.8)	164 (24.0)	
Yes	3273 (51.2)	2754 (48.2)	519 (76.0)	
Hyperlipidemia (N, %)				<0.001
No	3566 (55.8)	3262 (57.1)	304 (44.5)	
Yes	2829 (44.2)	2450 (42.9)	379 (55.5)	
Diabetes mellitus (N, %)				<0.001
No	5370 (84.0)	4880 (85.4)	490 (71.7)	
Yes	1025 (16.0)	832 (14.6)	193 (28.3)	
HEI-2015 (mean (SD))	49.5 (14.6)	49.8 (14.6)	53.0 (14.3)	<0.001
HEI-2015 quartile (N, %)				
Q1 (9.3–39.6)	1599 (25.0)	1473 (25.8)	126 (18.4)	<0.001
Q2 (39.6–49.5)	1599 (25.0)	1447 (25.3)	152 (22.3)	
Q3 (49.5–60.2)	1598 (25.0)	1412 (24.7)	186 (27.2)	
Q4 (60.2–96.1)	1599 (25.0)	1380 (24.2)	219 (32.1)	

**Table 2:** Association of HEI-2015 with cataract.

	Model 1 ^a^	Model 2 ^b^	Model 3 ^c^
HEI-2015	1.019 (1.014–1.024), <0.001	0.991 (0.984–0.997), 0.002	0.991 (0.984–0.997), 0.006
HEI-2015 quartile			
Q1	Ref	Ref	Ref
Q2	1.197 (0.932–1.539), 0.160	0.857 (0.633–1.162), 0.320	0.856 (0.630–1.164), 0.320
Q3	1.586 (1.251–2.016), <0.001	0.838 (0.627–1.121), 0.232	0.841 (0.630–1.131), 0.251
Q4	2.121 (1.692–2.671), <0.001	0.751 (0.569–0.940), 0.044	0.739 (0.559–0.980), 0.035

^a^ Non-adjusted model adjusted for none. ^b^ Minimally-adjusted model adjusted for gender, age, and race. ^c^ Fully-adjusted model adjusted for all covariates.

**Table 3:** Association of HEI-2015 and cataract using PSW-weighted regression.

	Model 1 ^a^	Model 2 ^b^	Model 3 ^c^
HEI-2015	0.991 (0.985–0.996), <0.001	0.992 (0.984–0.996), 0.002	0.990 (0.984–0.995), 0.002
HEI-2015 quartile			
Q1	Ref	Ref	Ref
Q2	0.770 (0.625–0.947), 0.014	0.824 (0.637–1.064), 0.139	0.824 (0.634–1.068), 0.144
Q3	0.760 (0.616–0.936), 0.010	0.779 (0.601–1.008), 0.058	0.780 (0.600–1.012), 0.062
Q4	0.748 (0.605–0.923), 0.007	0.747 (0.576–0.967), 0.027	0.744 (0.572–0.967), 0.027

^a^ Non-adjusted model adjusted for none. ^b^ Minimally-adjusted model adjusted for gender, age, race. ^c^ Fully-adjusted model adjusted for all covariates.

**Table 4:** Associations of HEI-2015 components with cataract.

HEI-2015 Component Score	Model 1 ^a^	Model 2 ^b^	Model 3 ^c^
Adequacy component score			
Total fruits	1.135 (1.093–1.179), **<0.001**	0.939 (0.895–0.984), **0.008**	0.947 (0.903–0.993), **0.027**
Whole fruits	1.114 (1.076–1.153), **<0.001**	0.945 (0.905–0.985), **0.009**	0.948 (0.907–0.991), **0.016**
Total vegetables	1.094 (1.042–1.149), **<0.001**	0.991 (0.936–1.050), 0.763	0.985 (0.930–1.044), 0.610
Greens and beans	0.981 (0.943–1.020), 0.349	0.990 (0.953–1.046), 0.965	1.005 (0.959–1.054), 0.820
Total protein foods	0.939 (0.883–0.998), **0.042**	0.986 (0.915–1.064), 0.718	0.979 (0.907–1.057), 0.581
Seafood and plant proteins	1.002 (0.967–1.040), 0.870	0.974 (0.933–1.017), 0.239	0.983 (0.941–1.027), 0.446
Whole grains	1.076 (1.051–1.102), **<0.001**	0.968 (0.940–0.997), **0.029**	0.966 (0.937–0.995), **0.024**
Dairy	1.030 (1.006–1.054), **0.013**	0.981 (0.953–1.010), 0.198	0.981 (0.953–1.010), 0.201
Fatty acids	0.999 (0.977–1.021), 0.941	1.003 (0.977–1.030), 0.806	1.004 (0.978–1.031), 0.752
Moderation component score			
Sodium	0.975 (0.954–0.997), 0.252	0.972 (0.947–0.997), **0.031**	0.979 (0.954–1.005), 0.113
Refined grains	0.995 (0.974–1.017), 0.672	0.953 (0.927–0.979), **<0.001**	0.958 (0.932–0.985), **0.002**
Added sugars	0.987 (0.968–1.006), 0.071	0.964 (0.929–1.000), 0.050	0.971 (0.916–1.026), 0.485
Saturated fats	1.034 (1.021–1.048), **<0.001**	1.003 (0.987–1.019), 0.696	1.001 (0.984–1.017), 0.935

^a^ Non-adjusted model adjusted for none. ^b^ Minimally-adjusted model adjusted for gender, age, race. ^c^ Fully-adjusted model adjusted for all covariates.

## Data Availability

The datasets used in the current study are available on the NHANES website: https://www.cdc.gov/nchs/nhanes/ (accessed on 18 October 2022).

## Supplementary Materials

The following supporting information can be downloaded from https://www.mdpi.com/article/10.3390/nu15010098/s1. Table S1: Characteristics of participants from NHANES 2005–2008 before propensity score weighting; Table S2: Characteristics of participants from NHANES 2005–2008 after propensity score weighting.
