# Supplementary material for: Association between Healthy Eating Index-2015 and Age-Related Cataract in American Adults: A Cross-Sectional Study of NHANES 2005–2008
Source: Nutrients. 2022 Dec 25;15(1):98. doi: 10.3390/nu15010098 (PMC9823857; doi:10.3390/nu15010098)
Supplement: Supplementary file 1 [file nutrients-15-00098-s001.zip › nutrients-2064283-supplementary.pdf]

Supplementary Materials

Table S1. Characteristics of participants from NHANES 2005-2008 before propensity score weighting.

Table S2. Characteristics of participants from NHANES 2005-2008 after propensity score weighting.

Table S1. Characteristics of participants from NHANES 2005-2008 before propensity score weighting.

	**HEI-2015 Q1 (9.3-39.6)**	**HEI-2015 Q2 (39.6-49.5)**	**HEI-2015 Q3 (49.5-60.2)**	**HEI-2015 Q4 (60.2-96.1)**	**P value**	**SMD**
Number	1599 (25.0)	1599 (25.0)	1598 (25.0)	1599 (25.0)		
Gender (N, %)					**<0.001**	0.103
Male	816 (51.0)	839 (52.5)	769 (48.1)	691 (43.2)		
Female	783 (49.0)	760 (47.5)	829 (51.9)	908 (56.8)		
Age (years, mean (SD))	50.22 (14.57)	52.84 (14.88)	55.17 (15.52)	58.37 (15.15)	**<0.001**	0.298
Race (N, %)					**<0.001**	0.142
Non-Hispanic White	835 (52.2)	780 (48.8)	848 (53.1)	870 (54.4)		
Non-Hispanic Black	394 (24.6)	371 (23.2)	288 (18.0)	292 (18.3)		
Mexican American	230 (14.4)	276 (17.3)	304 (19.0)	251 (15.7)		
Other	140 (8.8)	172 (10.8)	158 (9.9)	186 (11.6)		
Education (N, %)					**<0.001**	0.092
Less than high school	480 (30.0)	446 (27.9)	430 (26.9)	356 (22.3)		
High school or above	1119 (70.0)	1153 (72.1)	1168 (73.1)	1243 (77.7)		
Marital status (N, %)					**0.001**	0.074
Unmarried or other	623 (39.0)	560 (35.0)	518 (32.4)	535 (33.5)		
Married or living with partner	976 (61.0)	1039 (65.0)	1080 (67.6)	1064 (66.5)		
Poverty (N, %)					**<0.001**	0.094
Below poverty	299 (18.7)	267 (16.7)	258 (16.1)	194 (12.1)		
Poverty or above	1300 (81.3)	1332 (83.3)	1340 (83.9)	1405 (87.9)		
BMI (N, %)					**<0.001**	0.102
<18.5	30 (1.9)	18 (1.1)	11 (0.7)	24 (1.5)		
18.5~25	364 (22.8)	379 (23.7)	418 (26.2)	456 (28.5)		
≥25	1205 (75.4)	1202 (75.2)	1169 (73.2)	1119 (70.0)		
Alcohol usage (N, %)					**<0.001**	0.110
Lifetime abstainer	209 (13.1)	216 (13.5)	242 (15.1)	303 (18.9)		

Former drinker	273 (17.1)	280 (17.5)	268 (16.8)	258 (16.1)		
Current drinker ≤3 drinks/week	704 (44.0)	658 (41.2)	650 (40.7)	674 (42.2)		
Current drinker >3 drinks/week	413 (25.8)	445 (27.8)	438 (27.4)	364 (22.8)		
Smoking (N, %)					**<0.001**	0.334
Never smoke	703 (44.0)	797 (49.8)	849 (53.1)	956 (59.8)		
Former smoker	391 (24.5)	437 (27.3)	510 (31.9)	504 (31.5)		
Current smoker	505 (31.6)	365 (22.8)	239 (15.0)	139 (8.7)		
Hypertension (N, %)					0.071	0.047
No	797 (49.8)	780 (48.8)	807 (50.5)	738 (46.2)		
Yes	802 (50.2)	819 (51.2)	791 (49.5)	861 (53.8)		
Hyperlipidemia (N, %)					**0.004**	0.065
No	900 (56.3)	936 (58.5)	895 (56.0)	835 (52.2)		
Yes	699 (43.7)	663 (41.5)	703 (44.0)	764 (47.8)		
Diabetes mellitus (N, %)					**0.003**	0.069
No	1383 (86.5)	1348 (84.3)	1332 (83.4)	1307 (81.7)		
Yes	216 (13.5)	251 (15.7)	266 (16.6)	292 (18.3)		
Cataract (N, %)					**<0.001**	0.106
No	1473 (92.1)	1447 (90.5)	1412 (88.4)	1380 (86.3)		
Yes	126 (7.9)	152 (9.5)	186 (11.6)	219 (13.7)		

Table S2. Characteristics of participants from NHANES 2005-2008 after propensity score weighting.

	HEI-2015 Q1 (9.3-39.6)	HEI-2015 Q2 (39.6-49.5)	HEI-2015 Q3 (49.5-60.2)	HEI-2015 Q4 (60.2-96.1)	P value	SMD
Number	1621.0	1602.7	1589.2	1566.6		
Gender (N, %)					0.180	0.042
Male	766.3 (47.3)	822.1 (51.3)	777.6 (48.9)	757.0 (48.3)		
Female	854.7 (52.7)	780.6 (48.7)	811.5 (51.1)	809.6 (51.7)		
Age (years, mean (SD))	54.56 (15.79)	54.15 (15.22)	54.25 (15.55)	54.33 (15.09)	0.927	0.014
Race (N, %)					0.169	0.074
Non-Hispanic White	861.0 (53.1)	791.3 (49.4)	833.8 (52.5)	804.1 (51.3)		
Non-Hispanic Black	343.8 (21.2)	353.9 (22.1)	302.1 (19.0)	347.3 (22.2)		
Mexican American	260.5 (16.1)	278.3 (17.4)	300.9 (18.9)	257.1 (16.4)		
Other	155.8 (9.6)	179.1 (11.2)	152.3 (9.6)	158.0 (10.1)		
Education (N, %)					0.466	0.031
Less than high school	422.7 (26.1)	429.7 (26.8)	445.7 (28.0)	399.7 (25.5)		
High school or above	1198.3 (73.9)	1172.9 (73.2)	1143.4 (72.0)	1166.9 (74.5)		
Marital status (N, %)					0.361	0.035
Unmarried or other	586.7 (36.2)	547.4 (34.2)	525.5 (33.1)	546.7 (34.9)		
Married or living with partner	1055.2 (65.8)	1039 (65.0)	1063.7 (66.9)	1019.9 (65.1)		
Poverty (N, %)					0.362	0.036
Below poverty	242.3 (14.9)	256.1 (16.0)	267.2 (16.8)	228.4 (14.6)		
Poverty or above	1378.7 (85.1)	1346.5 (84.0)	1321.9 (83.2)	1338.2 (85.4)		
BMI (N, %)					0.128	0.064
<18.5	30.4 (1.9)	17.0 (1.1)	10.5 (0.7)	23.4 (1.5)		
18.5~25	394.3 (24.3)	399.3 (24.9)	410.1 (25.8)	380.2 (24.3)		
≥25	1196.4 (73.8)	1186.4 (74.0)	1168.6 (73.5)	1163.0 (74.2)		
Alcohol usage (N, %)					0.228	0.068
Lifetime abstainer	261.6 (16.1)	229.9 (14.3)	232.2 (14.6)	249.4 (15.9)		

Former drinker	263.9 (16.3)	278.7 (17.4)	265.9 (16.7)	259.8 (16.6)		
Current drinker ≤3 drinks/week	695.1 (42.9)	653.3 (40.8)	650.4 (40.9)	686.8 (43.8)		
Current drinker >3 drinks/week	400.4 (24.7)	440.8 (27.5)	440.7 (27.7)	370.6 (23.7)		
Smoking (N, %)					0.333	0.056
Never smoke	840.0 (51.8)	824.1 (51.4)	809.0 (50.9)	835.1 (53.3)		
Former smoker	464.1 (28.6)	446.5 (27.9)	485.1 (30.5)	459.7 (29.3)		
Current smoker	317.0 (19.6)	332.1 (20.7)	295.0 (18.6)	271.8 (17.3)		
Hypertension (N, %)					0.327	0.031
No	774.7 (47.8)	769.8 (48.0)	808.1 (50.9)	754.6 (48.2)		
Yes	846.4 (52.2)	832.8 (52.0)	781.1 (49.1)	812.0 (51.8)		
Hyperlipidemia (N, %)					0.077	0.051
No	862.4 (53.2)	921.3 (57.5)	900.8 (56.7)	850.6 (54.3)		
Yes	758.6 (46.8)	681.4 (42.5)	688.3 (43.3)	716.0 (45.7)		
Diabetes mellitus (N, %)					0.619	0.026
No	1365.2 (84.2)	1338.8 (83.5)	1331.9 (83.8)	1290.5 (82.4)		
Yes	255.9 (15.8)	263.9 (16.5)	257.2 (16.2)	276.1 (17.6)		
Cataract (N, %)					0.100	0.047
No	1405.8 (86.7)	1424.7 (88.9)	1415.9 (89.1)	1404.9 (89.7)		
Yes	215.2 (13.3)	178.0 (11.1)	173.3 (10.9)	161.7 (10.3)		
